# Exploration of differential responses to FODMAPs and gluten in people with irritable bowel syndrome- a double-blind randomized cross-over challenge study

**DOI:** 10.1007/s11306-023-02083-x

**Published:** 2024-02-12

**Authors:** Elise Nordin, Rikard Landberg, Per M. Hellström, Carl Brunius

**Affiliations:** 1 Department of Life Sciences, Division of Food and Nutrition Science, Chalmers University of Technology, 412 96 Gothenburg, Sweden; 2 Department of Medical Sciences, Gastroenterology/Hepatology, Uppsala University, 75185 Uppsala, Sweden

**Keywords:** Irritable bowel syndrome, Clinical trial, Double-blind, FODMAPs, Gluten, Personalized nutrition, Metabotyping, Differential response, Precision health

## Abstract

**Introduction:**

There is large variation in response to diet in irritable bowel syndrome (IBS) and determinants for differential response are poorly understood.

**Objectives:**

Our aim was to investigate differential clinical and molecular responses to provocation with fermentable oligo-, di-, monosaccharides, and polyols (FODMAPs) and gluten in individuals with IBS.

**Methods:**

Data were used from a crossover study with week-long interventions with either FODMAPs, gluten or placebo. The study also included a rapid provocation test. Molecular data consisted of fecal microbiota, short chain fatty acids, and untargeted plasma metabolomics. IBS symptoms were evaluated with the IBS severity scoring system. IBS symptoms were modelled against molecular and baseline questionnaire data, using Random Forest (RF; regression and clustering), Parallel Factor Analysis (PARAFAC), and univariate methods.

**Results:**

Regression and classification RF models were in general of low predictive power (Q^2^ ≤ 0.22, classification rate < 0.73). Out of 864 clustering models, only 2 had significant associations to clusters (0.69 < CR < 0.73, p < 0.05), but with no associations to baseline clinical measures. Similarly, PARAFAC revealed no clear association between metabolome data and IBS symptoms.

**Conclusion:**

Differential IBS responses to FODMAPs or gluten exposures could not be explained from clinical and molecular data despite extensive exploration with different data analytical approaches. The trial is registered at www.clinicaltrials.gov as NCT03653689 31/08/2018.

**Supplementary Information:**

The online version contains supplementary material available at 10.1007/s11306-023-02083-x.

## Introduction

Irritable bowel syndrome (IBS) is a complex condition characterized by recurrent abdominal pain associated with abnormal bowel habits (Lacy et al., 2016). The etiology of IBS is unknown; however, the pathogenesis is believed to be due to disturbed gut microbiota, visceral hypersensitivity, brain-gut interactions, intestinal inflammation, and psychosocial factors (Delvaux, 2002; Holtmann et al., 2016; Tanaka et al., 2011). Integrated care is consequently recommended, including dietary modifications, behavioral therapy, and medication (Chey et al., 2021).

Diet is considered a main cause of symptoms in IBS (Chey et al., 2021). Especially fermentable oligo-, di-, monosaccharides, and polyols (FODMAPs) are of major concern since they are not absorbed, causing intestinal osmotic load and bacterial fermentation. This in turn leads to increase of intestinal water and gas production, believed to cause symptoms such as abdominal pain and gas (Staudacher & Whelan, 2017). Hence, a low FODMAP diet is commonly followed in subjects with IBS (Dionne et al., 2018). Gluten has also been considered a major culprit, although a mechanistic rationale is lacking (Biesiekierski & Iven, 2015). It is well known that individuals respond differently to similar food intake (Zeevi et al., 2015), and personalization of dietary advice has been proposed as potentially more effective compared to general recommendations (Celis-Morales et al., 2017; Curtis et al., 2012). Finding objective biomarkers reflecting personalized response/non-response to dietary stressors of IBS has the potential to improve prevention of symptoms (Biesiekierski & Iven, 2015; Dimidi & Whelan, 2020; Hellström & Benno, 2019). However, evidence underpinning such personalization is still lacking. In addition, unnecessary exclusion should be avoided since FODMAPs constitute dietary fibers which are part of a healthy diet in line with dietary guidelines (Anderson et al., 2009; Blomhoff et al., 2023).

Several studies have attempted to understand differential responses to a low FODMAP diet. Differentiation of individuals into response types has been based on gut microbiota (Bennet et al., 2018; Chumpitazi et al., 2015; Colomier et al., 2022; Valeur et al., 2018; Vervier et al., 2021; Zhang et al., 2021), metabolite patterns (Nybacka et al., 2021), colonic methane and SCFA production (Eetemadi & Tagkopoulos, 2021), hydrogen production (Schindler et al., 2021), fecal volatile organic compounds (Rossi et al., 2018), intake of FODMAP at baseline (Böhn et al., 2015), and psychological and nutritional factors (Colomier et al., 2022). However, results from these studies are inconsistent, and even in some cases point in opposite directions: For example, a higher proportion of saccharolytic bacteria at baseline has been observed in both response and non-response of IBS symptoms after dietary interventions (Bennet et al., 2018; Chumpitazi et al., 2015; Zhang et al., 2021). In addition, other studies have not been able to identify differential responses to FODMAPs (Halmos et al., 2015; Staudacher et al., 2020). Concerning gluten, one clinical trial with a gluten-free diet versus a gluten-containing diet reported that response to the gluten-free diet could be predicted by the metabolite profile at baseline, identification of metabolites was not presented. However, the study was underpowered (Algera et al., 2022). To date, there are no generally accepted recommendations for tailored nutritional advice in IBS (Bennet et al., 2020; Drossman, 2016).

There is currently also no universal data-analytical framework for identification of differential response to dietary interventions. Both univariate (Böhn et al., 2015; Colomier et al., 2022; Halmos et al., 2015; Schindler et al., 2021; Valeur et al., 2018) and machine learning algorithms (Algera et al., 2022; Bennet et al., 2018; Chumpitazi et al., 2015; Nybacka et al., 2021; Rossi et al., 2018; Staudacher et al., 2020; Vervier et al., 2021; Zhang et al., 2021) have been applied in IBS and dietary trials, using the predictors mentioned above. Robust machine learning algorithms (Saccenti et al., 2014) could overcome the issue with multiple testing in univariate models, known to be problematic in analyzing large-scale omics data (Saccenti et al., 2014). Machine learning regression models have been used in IBS studies, however not with sufficient safeguards to minimize overfitting, such as from e.g. repeated double cross-validation (Filzmoser et al., 2009), and results may consequently be overly optimistic. Another approach, shown successful in previous studies (Hillesheim & Brennan, 2020), but not performed in IBS and dietary trials, is the grouping of individuals based on similarities in metabolism or metabolic regulation within group but differences between groups, i.e. metabotypes (Ciara Morris et al., 2013; Nicholson et al., 2012; Palmnäs et al., 2020), and relate such metabotypes to an outcome. Moreover, when studying metabolic pattern in time series data, methods to capture the dynamics of measured features over time and relating them to an outcome could be particularly useful. Parallel Factor Analysis (PARAFAC) (Bro, 1997) is a method well suited for such analysis but has previously not been used in IBS. Hence, these methods could be applied for new approaches and more robust modelling of differential responses.

We conducted a double-blind, randomized, controlled cross-over study with provocations of FODMAPs, gluten and placebo in subjects with IBS. The symptomatic response and effect on the fecal microbiota and the metabolome have previously been published (Nordin et al., 2022; Nordin, Hellström, Dicksved, et al. 2023; Nordin et al., 2023). In brief, the symptomatic response was measured with irritable bowel syndrome severity scoring system (IBS-SSS). At the treatment level, FODMAPs caused more severe symptoms (mean IBS-SSS 240 [95% CI 222, 257]) compared to placebo (198 [180, 215]; p = 0.00056), while there was no difference between gluten (208 [190, 226]) and placebo (p = 1.0). Concerning effects on the fecal microbiota, fecal and plasma short chain fatty acids (SCFAs) and the untargeted plasma metabolome, FODMAPs led to an elevation in fecal saccharolytic bacteria, phenolic-derived metabolites, and 3-indolepropionate, while concurrently causing a reduction in plasma isobutyrate and bile acids. The introduction of gluten resulted in a decrease in fecal isovalerate and brought minor alterations in carnitine derivatives, plasma fatty acids, and CoA. Regarding FODMAPs, there were modest correlations identified between the microbiota and phenolic-derived metabolites, as well as 3-indolepropionate, which have previously been associated with improved metabolic health and decreased inflammation (Roowi et al., 2009; Schär et al., 2018; Tuomainen et al., 2018). However, the correlations between molecular data and symptoms of IBS were found to be weak.

There was a large inter-individual variation in response to the interventions (Nordin et al., 2022) and we hypothesized that differential IBS responses to FODMAP intake could be related to molecular and questionnaire data, since FODMAPs are fermented by the gut microbiota (Hill et al., 2017), also affecting the metabolome (Subramanian et al., 2020), and IBS symptoms are considered to relate to gut microbiota (Chey & Menees, 2018). Moreover, since FODMAPs are rapidly fermented (Staudacher & Whelan, 2017), we hypothesized that characteristic phenotypic responses could be generated already after a rapid provocation test. We did not have a similar clear hypothesis for differential response to gluten, but due to the variability in response to treatment (Nordin et al., 2022), it was similarly investigated.

To address our hypotheses, we investigated whether differential responses could be related to a rapid pre-intervention provocation test containing both FODMAPs and gluten. Moreover, we investigated whether differential responses based on recorded IBS symptoms from week-long FODMAP and gluten provocations could be related to molecular phenotype data (including fecal microbiota, SCFAs, and metabolomics measurements), and questionnaire data at baseline. Furthermore, since there is no established universally accepted data analytical approach to identify differential responses (Palmnäs et al., 2020), a wide range of methodological approaches were explored to identify determinants for individual response or molecular subtypes, which may be applicable in a wider scope of health-related research.

## Methods

### Study design

**Fig. 1:**
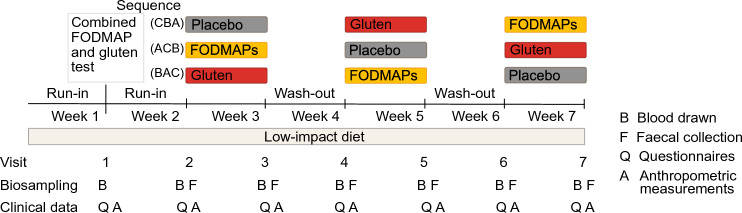
Study design of the three-way double-blind, randomized controlled cross-over study with interventions of FODMAP, gluten and placebo in people with IBS. Participants were randomized to the sequences CBA, ACB and BAC (A = FODMAPs, B = Gluten, C = Placebo). The figure is modified from Nordin et al. (Nordin et al., 2022)

A randomized, double-blind, placebo-controlled three-way crossover study was conducted in Uppsala, Sweden in September 2018 – June 2019, as described elsewhere (Nordin et al., 2022). Briefly, the study included 110 participants with moderate to severe IBS, of which 90 women and 13 men, aged 46 ± 14 years, and with BMI 24 ± 4 kg/m^2^ finished the trial. At the screening occasion, participants were diagnosed according to the Rome IV criteria (abdominal pain at least one day per week associated with two or more of the following criteria: related to defecation, associated with a change in frequency of stool or associated with a change in form (appearance) of stool. The criteria had to have been fulfilled for the last 3 months with symptom onset at least 6 months before screening) and subtyped into constipation (n = 29), diarrhea (n = 35) or mixed (n = 39). The severity of IBS was diagnosed with IBS-SSS (see Sect. 2.3). During the seven weeks of the trial, participants followed a so-called low-impact diet, excluding gluten and consuming minimal amount of FODMAPs, guided by dieticians specialized in IBS. After one week of low-impact diet, to test the hypothesis that molecular responses to a one-dose provocation could be indicative of symptoms during prolonged intervention, participants came to the clinic for a combined FODMAP and gluten challenge test, where blood samples and measurements were collected for four hours (visit 1). Following another week with low-impact diet, i.e. at visit 2, participants started one-week long interventions with FODMAP, gluten, and placebo exposures, with one week washout in-between. Participants and study personnel conducting the clinical trial were blinded. Questionnaires and fecal samples were handed in at visits 2–7, reflecting the preceding study week, and anthropometric measures and blood samples were collected. Oral and written informed consent was obtained from all participants before initiation of the trial. Participants were randomized in blocks of 12 into the sequences CBA, ACB, and BAC (A = FODMAPs, B = Gluten, and C = Placebo). Randomization of participants was performed by personnel not involved in the study. The allocation sequence was delivered to the study site one to three days before participants initiated the study. The blinding was broken when the trial was finished. The study was approved by the Ethics Review Board, Uppsala (2018/159) and registered at www.clinicaltrials.gov as NCT03653689 31/08/2018. The study was conducted according to the ethical principles of the Helsinki declaration. The study design is visually presented in Fig. 1, a flow chart of the participants during the trial is presented in **Supplementary Fig. 1**. The symptomatic response to the interventions has previously been published (Nordin et al., 2022). A table of the main results and a graph of the inter-individual variability of the response to the interventions are presented in **Supplementary Table 1** and **Supplementary Fig. 2**.

### Intervention foods

The combined FODMAP and gluten challenge at visit 1 was served as a cake containing 19.5 g fructose, 15.7 g lactose, 7 g fructo-oligosaccharides, 1.5 g galacto-oligosaccharides, 4.5 g sorbitol, and 1.8 g mannitol and 17.3 g gluten. Details about the food products [42] are presented in **Supplementary Table 2 and 3**. The daily dose of the week-long interventions with FODMAP or gluten corresponded to the respective FODMAP and gluten doses in the cake (in powder form), however divided into three servings per day, with rice porridge as the vehicle. The placebo intervention consisted of 18 g sucrose, similarly served. The doses of FODMAPs were calculated as 150% of the daily intake in an Australian population (Halmos et al., 2014) except for lactose and gluten, which were based on 150% of the intake in the Swedish population (Amcoff et al., 2012).

### Anthropometric data and questionnaires

At the screening occasion, measurement of blood pressure, waist circumference and weight were collected, and participants noted duration of IBS and if the onset was associated with a gastrointestinal infection or antibiotics use. Several questionnaires were filled out: IBS-SSS (Francis et al., 1997), Short Form 36 (SF-36v2 (Ware & Sherbourne, 1992), reflecting health and wellbeing), a demographic questionnaire (age, gender, ethnicity, educational and income level), Baecke’s physical activity questionnaire (Ono et al., 2007) and a validated food frequency questionnaire (Messerer et al., 2004) (energy adjusted with the residual method (Willet and Stampfer 1986) reflecting the dietary intake during the last year. IBS-SSS was the main questionnaire evaluating IBS symptoms in our trial. It is a validated questionnaire including the items: severity of abdominal pain, frequency of abdominal pain, abdominal distension, dissatisfaction with bowel habits and interference with life estimated on a visual analog scale (0–100). A composite total IBS score is reported between 0–500. IBS-SSS score < 175 is considered mild, ≥ 175 to ≤ 300 moderate and > 300 severe IBS. Of note, we used the questionnaire to evaluate IBS symptoms for 7 days, instead of the 10 days it has been developed for.

### Biological samples

Participants collected a fecal sample as close to each of the visits 2–7 as possible, preferably the last day before the visit, stored it at -20 °C in their home freezer until transportation to the clinic where samples were stored at -20 °C at most one week, after which they were transferred to -80 °C until analysis. Fecal microbiota composition was analyzed with the 16S rRNA method. In brief, DNA was extracted, the V3-V4 region was amplified with primers and sequencing libraries were generated, and sequence variants were compared to reference databases. After data processing, 132 amplicon sequence variant aggregated at genus level were included; details described elsewhere (Nordin E et al., 2023c).

During the provocation test, blood samples were drawn at -10, 0, 10, 20, 30, 90, 150, and 240 min. Thereafter, blood samples were collected at each visit. Directly after blood draw, samples were put on cold blocks and centrifuged within 30 min, stored in the clinic at −20 °C at most for one week, and thereafter transferred to -80 °C until analysis.

The SCFAs formate, acetate, propionate, butyrate, isobutyrate, valerate, isovalerate, the SCFA analogues caproate and succinate were analyzed in feces and plasma from the intervention weeks 2–7. Fecal samples were freeze dried and 20 mg was diluted with 1 mL LiChrosolv water, shaken and centrifuged. Samples were analyzed as described by Han et al. (Han et al., 2015) with addition of a quenching step (manuscript in preparation). In brief, for each batch, feces and plasma samples together with blanks and quality control samples (10 µL of each sample) were mixed with 10 µL 12C 3-nitrophenylhydrazine (3-NPH) and 10 µL N-(3-Dimethylaminopropyl)-N-ethylcarbidiimide hydrochloride (EDC-6), and 60 µL 75% methanol (3-NPH serves as the derivatizing reagent and EDC-6 as a coupling reagent). Samples were thereafter shaken and centrifuged. The reaction was quenched by 10 µL quinic acid, shaken and centrifuged, and an internal standard was added to the sample and analyzed with Q-TRAP QqQ-MS, details described elsewhere (Nordin et al., 2023d).

Untargeted metabolomics was analyzed in plasma samples from the rapid provocation test and for the intervention weeks 2–7. Briefly, plasma was mixed together with cold acetonitrile shaken, centrifuged, and filtered. Quality control samples were included in each batch. Samples were analyzed using a UHPLC-qTOF-MS system. For the intervention weeks, metabolomics data included 8618 features. Further details including preprocessing of metabolomics data for the intervention weeks is described elsewhere (Nordin et al., 2023b). Corresponding information for the provocation test is presented in Supplementary Text 1. Henceforth, fecal microbiota and plasma metabolome will simply be referred as ‘microbiota’ and ‘metabolome’.

### Data analysis

Sample size was based on the assumption that we will be able to identify around 4 ± 1 metabotypes, and an approximate equal distribution between metabotypes (≥ 20 persons per metabotype). To allow dropouts, 110 participants were included, assuring 100 completers. This number was arbitrary set since there is no consensus of how to perform power calculations in machine learning. However, this is a high number of participants in comparison to previous nutritional interventions.

**Fig. 2:**
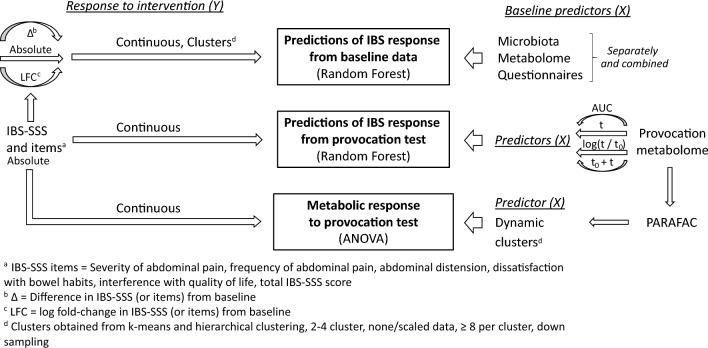
Machine learning was used to investigate differential responses to intervention (response) in relation to molecular and baseline clinical data (predictors). To investigate differential response at a continuous scale (i.e. individual responses), RF regression with IBS-SSS values as response was used. To evaluate potential associations at group level representing metabotypes, RF classification using clustered IBS-SSS responses as response was used. As predictors, the baseline microbiota, SCFAs, the metabolome, and baseline clinical data, were modelled separately and combined. RF regression with IBS-SSS values as response was used with the metabolome of the provocation test as predictor. Finally, unsupervised PARAFAC analysis of molecular data was performed to capture the dynamics of the metabolome for the 4 h of the provocation test. PARAFAC component scores were clustered and related to IBS response using ANOVA. Figure from doctoral thesis by Elise Nordin Chalmers, University Technology 2023 (Nordin, 2023). *ANOVA* analysis of variance, *AUC* Area under the curve, *IBS-SSS* Irritable bowel syndrome severity scoring system, *LFC* log fold change, *PARAFAC* Application of Parallel Factor Analysis, *RF* Random Forest, *SCFA* Short chain fatty acids, *t* timepoint −10 min (−10, 0, 10, 20, 30, 90, 150, 240 min)

A comprehensive methodological investigation was performed including several methods attempting to relate differential IBS responses to interventions in relation to omics and questionnaire-data from baseline and the provocation test. An overview of the structure of the statistical analysis is presented in Fig. 2. We first used machine learning (Random Forest, RF) to investigate differential responses to treatment reflected by IBS-SSS (response variable) in relation to molecular (microbiota, SCFAs, and metabolome), and baseline questionnaires data, separate or combined (predictor variables). RF was selected since it does not require specific variable distributions, scaling, or underlying linearity in associations between predictors and response. Furthermore, it is insensitive to scaling or transformations of the predictor variables and thus less sensitive to variable preprocessing schemes (Qi, 2012). Considering that machine learning methods are prone to overfitting (Shi et al., 2019), in combination with a high number of models investigated in this exploratory investigation, we performed RF modelling using the MUVR algorithm (Shi et al., 2019) (version 0.0.9), which performs repeated double cross-validation (Filzmoser et al., 2009).

This supervised RF analysis was performed using two main approaches: First, to investigate differential response at an individual level using RF regression with IBS-SSS values as response on a continuous scale. Second, to evaluate potential associations at a group level, corresponding to IBS-related metabotypes, using RF classification with clustered IBS-SSS responses as response. Both approaches were modelled using several different representations of response and predictor data to extensively explore potential associations between IBS response and different molecular predictors.

In addition, unsupervised analysis was performed to capture the overall dynamics of the metabolome for the 4 h of the provocation test. For this purpose, as a first step we used PARAFAC (Bro, 1997), which can be seen as an extension of a principal component analysis (PCA) into additional dimensions (in this case, individuals, metabolite features, and time points). In a second step, component scores from PARAFAC were clustered and further related to IBS response for the interventions using ANOVA. Due to the exploratory nature of the analyses, all models were run as per protocol. All analyses were performed in the programming language R (version 4.0.0).

#### Predictions of IBS response from baseline molecular data

### Response variables

In all analyses, we explored the differential response of IBS-SSS data from the interventions and not from the rapid provocation test as response variable. The total IBS-SSS score as well as its five separate items (severity of abdominal pain, frequency of abdominal pain, abdominal distension, dissatisfaction with bowel habits, and interference with life) were modelled as response variables, both as absolute values, as log fold change and difference compared to the preceding washout week, separately per intervention (FODMAPs, gluten, and placebo). The response variables were modelled both as continuous variables (i.e., the actual recorded response values) to represent associations at an individual level and as categories (i.e., groups of individuals from clustering) to represent potential metabotypes, as described above. In clustering, the measurements of each IBS-SSS item from all three treatment arms were condensed into a data frame. Clustering was performed as hierarchical clustering (Euclidean distance) and k-means clustering, using both scaled and non-scaled data, with 2–4 clusters, limited to clustering solutions with a minimum of 8 participants per cluster. In order not to bias classification analyses to the majority class, clusters were randomly downsampled to obtain equal number of individuals per cluster.

### Predictor variables

Differential responses to the interventions were then investigated in relation to baseline data as predictors, including all data collected at screening and microbiota, fecal and plasma SCFAs, and metabolome data collected at the beginning of the study (study visit 2). Data collected at screening were: IBS severity and subtype, duration of IBS, if IBS onset was associated with a gastrointestinal infection or antibiotic use, the questionnaires IBS-SSS, SF-36v2, the demographic questionnaire, Baecke’s physical activity questionnaire, food frequency questionnaire, and anthropometric measurements. In total, the questionnaires consisted of 124 variables. Data collected at screening is henceforth referred to as ‘baseline clinical data’.

Associations between response and predictor variables were modeled with the MUVR-RF algorithm (Shi et al., 2019). Predictors were first modelled separated by the different data types (microbiota, SCFAs, the metabolome, and baseline clinical data) not to dilute associations per data type with additional variables potentially contributing with noise. Predictor data were also used in combination to allow for implicit interaction between variables from different types. A priori limits of Q^2^ > 0.2 (for regression) or classification rate (CR) > 0.6 were used as filters to identify potentially informative models that were further analyzed by permutation tests (n = 100) to assure response was not due to overfitting. The a priori cutoffs were arbitrary selected to correspond to 20 percent better prediction than random chance. For clustering, downsampling can reduce the number of subjects included in the analysis. Models that had CR > 0.6 were therefore re-run 5 times (each time with a new random downsampling) together with permutation tests to ensure that findings were not due to overfitting. Average CR ± SD was reported together with p-value from the permutation tests. Clusters from classification models reaching the threshold (p_permutation_ < 0.05) were associated to baseline clinical data using ANOVA for continuous variables and Fisher’s test for categorical variables. Adjustment for multiple testing was performed according to the Benjamini–Hochberg false discovery rate procedure, considering FDR-adjusted p-value < 0.05 as significant.

#### Predictions of IBS response from molecular data of a provocation test

To investigate differential responses in relation to metabolite data of the provocation test, the IBS-SSS items from each intervention week (absolute values) were modelled as RF regressions using the dynamic metabolomics data from the provocation test as predictors. Metabolomics data were represented either by each time point during the provocation test (−10 = pre-test, 0, 10, 20, 30, 90, 150, and 240 min), area under the curve (AUC), log2(timepoint/pre-test) or by the combined data of pre-test and subsequent time points one by one. The data were comprehensively explored to discover any relevant associations corresponding to differential response. The stability of the metabolome during the provocation test was measured with intra class correlation with a two-way random-effect model (type = single measure, definition = absolute agreement).

#### Metabolic response to provocation (PARAFAC)

The metabolomics data from the provocation test were also explored using PARAFAC to investigate potential different dynamic patterns. Data were ordered into a tensor, missing values were imputed (matrix completion, R package eimpute version 0.1.1) and the data were preprocessed in two ways: (i) by scaling per metabolite to a standard deviation of one (Skantze et al., 2023) and; (ii) subtracting the global metabolite average per individual and scaling the data per metabolite feature to the root mean square of 1 (Bro & Smilde, 2003). PARAFAC was run with 2–20 components. Scores were clustered per component using hierarchical and k-means clustering (same settings as above) to represent potential metabotypes. For each model, ANOVA was run to evaluate a potential link to IBS symptoms (for each intervention and IBS-SSS item as absolute values).

## Results

In both the intervention and the provocation test models, 74 individuals were included in the analyses. The trial included 110 subjects, exclusions were due to: dropouts (n = 7), intake of probiotics (n = 1), antibiotics (n = 2), non-compliance (n = 7), deviations from inclusion criteria in BMI (n = 2), age (n = 2), lactose intolerance (n = 5), intake of symptom-mitigating pharmaceuticals (n = 2), and un-subtyped IBS (n = 14). Exclusion criteria overlapped for some individuals. Details are further described elsewhere (Nordin et al., 2022). No adverse events were reported during the clinical trial.

### Predictions of IBS response from baseline and provocation molecular data

When predicting intervention and IBS-SSS outcomes from baseline data with RF regression, using the microbiota, SCFAs, the metabolome, clinical data, or a combination as predictors, 270 models were run. However, only one model reached the a priori limit (Q^2^ > 0.2): total IBS-SSS score in the placebo arm using microbiota as predictors (Q^2^ = 0.22, p = 0.004). When analyzing clusters of response (representing metabotypes), out of 864 potential models, 429 models reached the requirement of ≥ 8 subjects per cluster. Of these, 12 reached the a priori limit for predictive performance (CR > 0.6) Analyses using data modelled as actual values is presented in Table 1. Modelled instead using difference or log fold change to the preceding washout week produced similar results (Supplementary Table 4). Only two of these models, both related to frequency of abdominal pain, reached significance at p < 0.05 (CR = 0.73 Table 1**;** CR = 0.69 **Supplementary Table 4**). However, none of the clusters associated with baseline clinical data. In addition, none of the 69 models predicting IBS response to the interventions (response) from rapid provocation test metabolite data (predictors) reached the a priori Q^2^ limit.

**Table 1:** Output for Random Forest classification modelling

IBS-SSS variable	Method	N clusters	Predictor data	CR[SD]	P_perm_
Total IBS-SSS score	Hclust	2	SCFAs	0.63 ± 0.05	0.12
Total IBS-SSS score	Hclust	2	Combination	0.63 ± 0.08	0.15
Frequency of abdominal pain	Kmeans	2	Metabolome	0.73 ± 0.02	0.006
Frequency of abdominal pain	Kmeans	2	Combination	0.69 ± 0.14	0.08

Each IBS-SSS item from all three treatment arms were condensed into a data frame before clustering (response) while the baseline microbiota, SCFAs, the metabolome, or a combination were used as predictors. The table shows models were CR > 0.6 and response variables were modelled as ‘absolute’ value and data were scaled. A complete presentation of all relevant models is presented in Supplementary Table 4

*CR* classification rate, *Hclust* hierarchical culstering, *IBS-SSS* – irritable bowel syndrome - severity scoring system, *perm* permutation, *SCFAs* short chain fatty acids

### Metabolic response to provocation (PARAFAC)

The metabolome was clearly perturbed by the rapid provocation test, since 79% of the metabolites had intra class correlation < 0.5 (**Supplementary Fig. 3**). Using hierarchical clustering on scaled data, 6978 out of in total 11,286 models reached the requirement of ≥ 8 subjects per cluster. Among these, 267 were nominally significant at p < 0.05, of which 87 related to FODMAPs, 71 to gluten and 109 to placebo, i.e. with no enrichment of associations to treatment groups vs placebo. Results were similar for all clustering approaches investigated (k-means and hierarchical using both scaled and non-scaled data (**Supplementary Table 5**). These models were further manually inspected to examine whether specific groups of individuals were reproducibly clustered in the different models, potentially representing robust metabotypes. However, no such robust participant clustering was observed.

## Discussion

To our knowledge, this is the first study aiming to unravel determinants of differential IBS responses to FODMAP and gluten provocation interventions from molecular data. The data-driven approaches for differential response analysis included machine learning-based predictions of IBS response from both baseline and provocation-related molecular data. Despite a comprehensive set of methods applied to explore IBS responses, including both regression and classification, predictors of differential response could not be established. Of note, one regression model and two classification models associated IBS outcomes to baseline molecular (omics) predictors. However, these models nonetheless likely did not correspond to actionable differential responders: First, the predictive power was not sufficiently high to warrant accurate subtyping or precision interventions. Second, even the use of repeated double cross-validation does not provide a complete safeguard against false positive discovery. Third, considering the large number of models run for each intervention and IBS-SSS item, true underlying associations would likely have produced a more pronounced enrichment of significant models. In fact, only a few associations were significant and none of the observed clustering models associated with phenotype traits measured by baseline clinical variables. Hence, the few significant models were not further investigated. In addition, we also applied a PARAFAC-based approach to capture metabolite dynamics over the duration of the provocation test to identify potential metabotype clusters that could be related to differential IBS-responses. Associations of IBS-SSS symptoms to the PARAFAC-based clusters were weak, and with no enrichment in associations for the FODMAP or gluten exposures compared to the placebo control intervention. This suggests that the immediate postprandial metabolite dynamics following exposure to FODMAP and gluten did not relate to IBS severity. Hence, no molecular basis for differential response could be identified based on microbiota, SCFAs, the metabolome, or clinical data; neither on individual level nor on group (metabotype) level. In the analyses no cutoff in response (IBS-SSS) was set, as a percentage of required change or the common cutoff with IBS-SSS, a change of 50 points (Francis et al., 1997). The reason was that using the actual response as a continuous variable has two important features: maintained power (from keeping all observations in the analysis, dichotomized models may require downsampling) and resolution (from relating predictor data to the degree of effect rather than dichotomized effect).

In contrast to our results and some previous studies (Halmos et al., 2015; Staudacher et al., 2020), most IBS studies have reported differential response related to microbiota composition, metabolite pattern, colonic methane, hydrogen and SCFA production, fecal volatile organic acids, intake of FODMAPs at baseline, psychological and nutritional factors at baseline (Bennet et al., 2018; Böhn et al., 2015; Chumpitazi et al., 2015; Colomier et al., 2022; Eetemadi & Tagkopoulos, 2021; Nybacka et al., 2021; Rossi et al., 2018; Schindler et al., 2021; Valeur et al., 2018; Vervier et al., 2021; Zhang et al., 2021). Of note is that most findings in previous studies have never been replicated, and some results have also been contradictory, such as saccharolytic genera being enriched in both response and non-response (Bennet et al., 2020; Chumpitazi et al., 2015; Zhang et al., 2021). It is likely that the inconsistencies in the literature relate to methodological issues in several of the performed IBS studies, including small sample size as well as data-analytical issues, including lack of robust methods to avoiding overfitting in machine learning modelling (Filzmoser et al., 2009).

There may be several reasons why no apparent differential responses to IBS symptoms could be detected in the present study. For example, potentially important molecular determinants may have not been measured and therefore not included in the analyses (Rubin & van der Laan, 2012). Psychological factors are important in IBS (Chey et al., 2021; Ohlsson, 2022) and may represent such unmeasured factors. Stratification of patients based on psychological markers is, according to Staudacher et al. (Staudacher et al., 2021), necessary for better understanding of the response to dietary treatment. In addition, as previously reported (Nordin et al., 2022), in the present trial, for the FODMAP, gluten and placebo intervention, there were no difference in increase of > 50 point in total IBS-SSS (46%, 37%, and 35%, respectively; p > 0.12), considered a clinically significant effect (Francis et al., 1997). The high nocebo response is in line with reported high nocebo responses in IBS challenge trials (Lembo, 2020; Li et al., 2022), further highlighting the challenges with psychological aspects. For the provocation test, there was no control and it was therefore not possible to properly evaluate the symptomatic response.

Concerning gluten, the general intervention effect was weak, both on IBS symptoms (Nordin et al., 2022), as well as on microbiota composition and SCFAs (Nordin E et al., 2023a), and the metabolome (Nordin E et al., 2023). At the same time, there was large interindividual variability in symptomatic response and therefore a potential to identify determinants thereof (Nordin et al., 2022), in line with previous studies (Molina-Infante & Carroccio, 2017; Skodje et al., 2018). However, we found no associations in the data to support this hypothesis. A lack of a clinical or molecular basis for differential response is likely related to similar reasons as for the FODMAP intervention, i.e. IBS being a heterogenous condition and important molecular determinants were not included. One previous study identified response to a gluten free diet versus a gluten-containing diet, but that study was underpowered for such a sub-analysis of differential response (Algera et al., 2022).

### Limitations and strengths

This study has several limitations: First, there was no true baseline value for the microbiota and the metabolome since the sampling was collected after initiation of the low impact diet. Second, although a wide set of baseline variables was included, important variables were lacking, e.g. psychological variables. Inclusion of such variables is strongly encouraged for future trials. Third, the use of sucrose as placebo could have potential issues since there are reported mutations in the sucrase-isomaltase gene in IBS (Henström et al., 2018). However, since there was no effect of placebo in this trial (by comparing to the wash-out weeks (Nordin et al., 2022), it does not seem to have had an important impact of the study. Fourth, the sex imbalance in recruitment makes the results mainly valid for women. Uneven distribution in recruitment of sex is a known issue in IBS studies and additional efforts need to be implemented in upcoming trials. Our study also has several strengths: The study was of large sample size, and in fact larger than any previous trial investigating differential responses in IBS. Importantly, the double-blind crossover design contributed objectivity. Moreover, a strength of the study was the large amount of data included at different time points and therefore potential to identify molecular markers to address differential response to treatment. Thereby also the potential to use different methodological approaches to identify differential response. Finally, the use of a double cross-validation regimen reduced overfitting and false positive discovery. Without these precautions more models would probably have generated significant but unsubstantiated associations.

## Conclusion

We performed the hitherto largest double-blind study with a comprehensive exploration with multiple analytical approaches to understand differential IBS responses to FODMAP and gluten exposure and mechanisms thereof. Yet, no explanations to differential responses were found. The complexity of IBS, including the fact that its pathophysiology is still unknown, are likely to contribute to the observed difficulties in subgrouping. Unmeasured baseline variables, such as psychological factors, may have carried important information for such subtyping. We encourage the application of molecular subtyping methodologies outlined here in other studies where differential responses to treatment are expected.

### Supplementary Information

Below is the link to the electronic supplementary material.

Supplementary file1 (PDF 352 KB)

## Data Availability

The datasets generated and analyzed during the current study are not publicly available due to privacy restrictions but are available from the corresponding author on reasonable request.

## Abbreviations

CR: Classification rate
FODMAPs: Fermentable oligo-di-monosaccharides and polyols
IBS: Irritable bowel syndrome
IBS-SSS: IBS severity scoring system
PARAFAC: Parallel factor analysis
SCFAs: Short chain fatty acids
