# Supplementary material for: Exploration of differential responses to FODMAPs and gluten in people with irritable bowel syndrome- a double-blind randomized cross-over challenge study
Source: Metabolomics. 2024 Feb 12;20(2):21. doi: 10.1007/s11306-023-02083-x (PMC10861383; doi:10.1007/s11306-023-02083-x)
Supplement: Supplementary file 1 — Supplementary file1 (PDF 352 KB) [file 11306_2023_2083_MOESM1_ESM.pdf]

Supplementary information

Exploration of differential responses to FODMAPs and gluten in people with irritable bowel syndrome- a double-blind randomized cross-over challenge study

Elise Nordin*[1], Rikard Landberg[1], Per M. Hellström[2], Carl Brunius[1]

[1] Department of Life Sciences, Division of Food and Nutrition Science
Chalmers University of Technology, 412 96 Gothenburg, Sweden

[2] Uppsala University, Department of Medical Sciences, Gastroenterology/Hepatology
75185 Uppsala, Sweden

*Correspondence author
E-mail: elise.nordin@chalmers.se (EN)

Supplementary Text 1.

The programming language R (version 3.6.1) was used for data pre-processing and statistical analysis. Data from the reversed-phase positive (RP) and reversed phase negative (RN) modes were processed separately. The R package XCMS[1] (version 3.6.1) was used for peak picking, retention time alignment, correspondence, and filling. The parameters noise, prefilter, snthresh, and bw were set manually after computer-assisted optimization: Parameters were optimized per batch with the R packages IPO[2] (version 1.10.0) and the batch averages were used to process all files. In total, 10827 metabolite features (peaks with a unique m/z ratios and retention time (RT)) were obtained for RP and 7579 for RN. Gap-filling was performed with the XCMS algorithm FillChromPeaksParam. After XCMS processing, 8 and 7 percent of the data was missing in RP and RN, respectively, and imputation was performed with an in-house Random Forest based algorithm, mvImpWrap() from https://gitlab.com/CarlBrunius/StatTools. Normalization of systematic drift within and between batches was performed with the R package batchCorr[3] (version 0.2.4). After normalization there were 9741 features for RP and 6909 for RN. Finally, isotopes, adducts and fragments assumed to derive from the same metabolite were grouped with the R packages RamClust[4] (version 1.1.0) resulting in 4610 features in positive mode and 3220 features in negative mode.

Parameters in XCMS

Reversed phase chromatography – Negative ionization (RN)

Peak picking:

CentWaveParam(peakwidth=c(8.6,58.4), noise=500, snthresh=10, ppm=17.3, mzdiff=0.00193, prefilter=c(3,3500), integrate=1)

Retention time correction:

PeakDensityParam(minfrac=0.95, bw=3, binSize=0.02)
peakGroupParam(minfrac=0.95,smooth=”loess”, span=0.4, family=”gaussian”)

Correspondance:

PeakDensityParam(minfrac=0.4, bw=1.5, binSize=0.02); FillChromPeaksParam(ppm=19, fixedRt=0.25, expand=0.1)

Reversed phase chromatography – Positive ionization (RP)

Peak picking:

Batch 1-10: CentWaveParam(peakwidth=c(8.6,48.8), noise=500, snthresh=10, ppm=19.05, mzdiff=-0.00223, prefilter=c(3,5000), integrate=1)

Batch 11: CentWaveParam(peakwidth=c(8.4,50), noise=500, snthresh=10, ppm=34, mzdiff= -0.0065, prefilter=c(3,1000), integrate=1)

Batch 1-10 and batch 11 were merge and thereafter followed the same parametrization procedure.

Retention time correction:

PeakDensityParam(minfrac=0.95, bw=3, binSize=0.02)
peakGroupParam(minfrac=0.95,smooth=”loess”, span=0.4, family=”gaussian”)

Correspondance:

PeakDensityParam(minfrac=0.4, bw=2, binSize=0.02); FillChromPeaksParam(ppm=22, fixedRt=2, expand=0.2)

Parameters used for RamClust

Parameters were manually optimized by visual inspection of extracted ion chromatograms of 20 randomly selected cluster, resulting in st=1.25, sr=0.35 in both modes. This resulted in 1018 clusters in RN and 1377 in RP and 2784 and 3439 singletons, respectively. For downstream analysis, the feature with highest intensity in each cluster was selected together with the singletons.

Supplementary Table 1. Total IBS-SSS score after interventions (FODMAPs, gluten, or placebo). Elevated scores indicate more severe symptoms.

	**FODMAPs**	**Gluten**	**Placebo**	**p-value**	**FODMAPs-Placebo**	**FODMAPs-Gluten**	**Gluten-Placebo**
Total IBS-SSS score	240 [9] (222, 257)	208 [9] (190, 226)	198 [9] (180, 215)	0.0023	42 [11] (20, 64) p=0.00056	32 [11] (10, 54) p=0.013	10 [11] (-11, 31) p=1.0
Severity of abdominal pain	35 [2] (31, 40)	34 [2] (29, 38)	32 [2] (27, 36)	1.0			
Frequency of abdominal pain	58 [4] (51, 65)	49 [4] (42, 55)	44 [3] (37, 51)	0.012	14 [4] (6, 22) p=0.0020	9 [4] (1, 17) p=0.072	5 [4] (3, 13) p=0.74
Abdominal distension	45 [2] (40, 49)	37 [2] (33, 42)	32 [2] (28, 37)	0.00025	13 [3] (7, 19) p < 0.0001	8 [3] (2, 14) p=0.023	5 [3] (-1, 11) p=0.25
Dissatisfaction with bowel habits	56 [2] (52, 60)	52 [2] (48, 56)	50 [2] (46, 54)	0.51			
Interference with quality of life	55 [2] (51, 59)	50 [2] (46, 54)	52 [2] (47, 56)	0.29			

Mixed linear models were used with intervention and period as fixed factors and participant as the random factor (total n=103). Data are presented as mean [SEM] (95% CI).

Abbreviations: FODMAPs, fermentable oligo-, di-, monosaccharides and polyols; IBS-SSS, irritable bowel syndrome severity scoring system.

Supplementary Table 2. Daily intake of rice porridge (3 servings per day) with FODMAPs, gluten, and placebo.

	Daily rice porridge intake			
	**Cake (g)**	**FODMAPs (g)**	**Gluten (g)**	**Placebo (g)**
Fructose	19.5	19.5	0	0
Lactose	15.7	15.7	0	0
FOS	7.0	7.0	0	0
GOS	1.5	1.5	0	0
Sorbitol	4.5	4.5	0	0
Mannitol	1.8	1.8	0	0
Gluten	17.3	0	17.3	0
Cocoa	4.0	0	0	0
Sucrose	0	0	0	18.0
Icing sugar	0	0	24.0	0
Rice flakes	0	78.0	78.0	78.0

Abbreviations: FODMAPs, fermentable oligo-, di-, monosaccharides and polyols; FOS, fructo-oligosaccharides; GOS, galacto-oligosaccharides.

Fructose (Minimum 99.5%, Engelhardt, Sweden, Caldic, Sweden)
Lactose (Minimum 99%, Engelhardt, Sweden, Caldic, Sweden)
FOS (97 ± 2%, Caldic, Sweden)
GOS (69%, plus 23% lactose, 5% glucose and galactose, FrieslandCampina Ingredients, Netherlands)
Sorbitol (minimum 97%), mannitol (minimum 98%, Roquette, France)
Gluten (78%, Lantmännen, Sweden)
Sucrose and icing sugar (Engelhardt, Sweden)
Cocoa (Fazer, Finland)
Rice flakes (Quaker, Orkla Foods Sverige AB, Sweden)

Supplementary Table 3. Nutritional contents of rice porridges (FODMAPs, gluten, and placebo).

	Cake		Rice porridge with (per 100 g)			Daily intake of rice porridge (3 servings) with		
	per 100 g	per serving	FODMAPs	Gluten	Placebo	FODMAPs	Gluten	Placebo
Energy (kcal)	349.1	275.4	397.6	401.2	397.6	492.7	472.9	372.7
Protein (g)	22.9	18.1	4.7	18.1	5.9	5.8	21.3	5.5
Ash (g)	0.6	0.5	0.3	0.4	0.4	0.4	0.5	0.4
Fat (g)	2.5	2.0	0.5	1.7	0.7	0.6	1.9	0.7
TC (g)	58.7	46.3	93.7	78.5	91.8	116.1	92.5	86.1
Fructose (g)	24.5	19.3	17.0	< 0.04	< 0.04	21.1	< 0.04	< 0.04
Lactose (g)	18.3	14.4	12.2	< 0.04	< 0.04	15.1	< 0.04	< 0.04
FOS (g)	8.7	6.9	4.7	0.4	0.3	5.8	0.5	0.2
GOS (g)	2.4	1.9	1.5	< 0.03	< 0.03	1.9	< 0.03	< 0.03
Sorbitol (g)	5.2	4.1	3.3	< 0.04	< 0.04	4.1	< 0.04	< 0.04
Mannitol (g)	2.1	1.7	1.4	< 0.04	< 0.04	1.7	< 0.04	< 0.04
DF (g)	1.6	1.2	0.9	1.3	1.1	1.1	1.6	1.0

Abbreviations: DF, dietary fiber; FODMAPs, fermentable oligo-, di-, monosaccharides and polyols; FOS, fructo-oligosaccharides; GOS, galacto-oligosaccharides; TC, total carbohydrates.

	Analysis	Method
Energy	Calculated by authors	1 g carbohydrates = 4 calories, 1 gram protein = 4 calories, 1 g fat = 9 calories
Protein	Eurofin	Nitrogen, determination in foods based on Kjeldahl (Nordic Committee on Food Analysis (NMKL) 6, 4th ed., 2003)
Ash	Eurofin	Ash, gravimetric determination in foods (NMKL 173, 2nd ed., 2005)
Fat	Eurofin	Fat, determination in foods. (NMKL 160, 1998)
TC	Calculated by authors	By difference: 10 (weight in grams) - [protein + fat + water + ash]
Fructose	Eurofin	the Association of Official Agricultural Chemists (AOAC) 982.14, mod.
Lactose	Eurofin	AOAC 982.14, mod.
FOS	Swedish University of Agricultural Sciences, Uppsala, Sweden	AOAC method 999.03
GOS	Eurofin	AOAC 2001.02
Sorbitol	Eurofin	High-performance liquid chromatography
Mannitol	Eurofin	High-performance liquid chromatography
DF	Swedish University of Agricultural Sciences, Uppsala, Sweden	AOAC method 994.13, with modifications by Andersson et al. (1999)

Supplementary Table 4. Output for Random Forest classification modelling. Each IBS-SSS item from all three treatment arms were condensed into a data frame before clustering (response) while the baseline microbiota, SCFAs, the metabolome, or a combination were used as predictors. Of 864 models, 12 reach the a priori limit for predictive performance but only two reached significant (CR > 0.6 and p < 0.05).

IBS-SSS variable	Data format	Data scaling	Method	N clusters	Predictor data	CR[SD]	Pperm
Total IBS-SSS score	Log2	Yes	Hclust	2	Microbiota	0.61±0.14	0.27
Total IBS-SSS score	Abs	Yes	Hclust	2	SCFAs	0.63±0.05	0.12
Total IBS-SSS score	Diff	Yes	Hclust	2	SCFAs	0.66±0.02	0.07
Total IBS-SSS score	Abs	Yes	Hclust	2	Combination	0.63±0.08	0.15
Frequency of abdominal pain	Diff	Yes	Kmeans	2	SCFAs	0.66±0.1	0.15
Frequency of abdominal pain	Abs	No	Kmeans	2	Metabolome	0.66±0.05	0.06
Frequency of abdominal pain	Log2	Yes	Hclust	2	Metabolome	0.65±0.1	0.21
Frequency of abdominal pain	Abs	Yes	Kmeans	2	Metabolome	0.73±0.02	0.006
Frequency of abdominal pain	Diff	Yes	Kmeans	2	Metabolome	0.69±0.05	0.03
Frequency of abdominal pain	Diff	No	Kmeans	2	Metabolome	0.63±0.05	0.07
Frequency of abdominal pain	Abs	Yes	Kmeans	2	Combination	0.69±0.14	0.08
Dissatisfaction with bowel habits	Diff	No	Kmeans	2	Microbiota	0.60±0.03	0.15

Abbreviations: Abs, data as absolute values; CR, classification rate; diff, data as difference between the intervention and the preceding washout week; Hclust, hierarchical clustering, IBS-SSS – irritable bowel syndrome - severity scoring system; perm, permutation; SCFAs, Short chain fatty acids

Supplementary Table 5. Distribution of observed associations between PARAFAC clusters and IBS-SSS items for the interventions FODMAPs, gluten, and placebo. Data were modelled with hierarchical clustering and k-means, using both scaled and non-scaled data. The results show that PARAFAC clusters did not relate to the FODMAP or gluten related IBS-SSS items to a greater extent than placebo.

	**Intervention**	**Number of significant models**	**Severity of abdominal pain**	**Frequency of abdominal pain**	**Abdom-inal distension**	**Dissatisfa-ction with bowel habits**	**Interfere-ence with quality of life**	**Total IBS-SSS score**
**Hclust, scaled data**	FODMAPs	87	14	16	16	15	10	16
	Gluten	71	9	13	16	15	6	12
	Placebo	109	12	22	22	15	17	21
**Hclust, non-scaled data**	FODMAPs	88	17	11	19	16	14	11
	Gluten	61	15	5	15	10	8	8
	Placebo	96	17	14	25	15	13	12
**Kmeans, scaled data**	FODMAPs	115	15	16	26	26	14	18
	Gluten	72	15	12	18	14	9	4
	Placebo	130	14	25	24	25	25	17
**Kmeans, non-scaled data**	FODMAPs	92	5	19	23	22	11	12
	Gluten	78	12	12	16	22	5	11
	Placebo	111	14	16	24	19	18	20

Abbreviations: FODMAPs, fermentable oligo-, di-, monosaccharides, and polyols; Hclust, hierarchical clustering; PARAFAC, application of parallel factor analysis; IBS-SSS, irritable bowel syndrome - severity scoring system

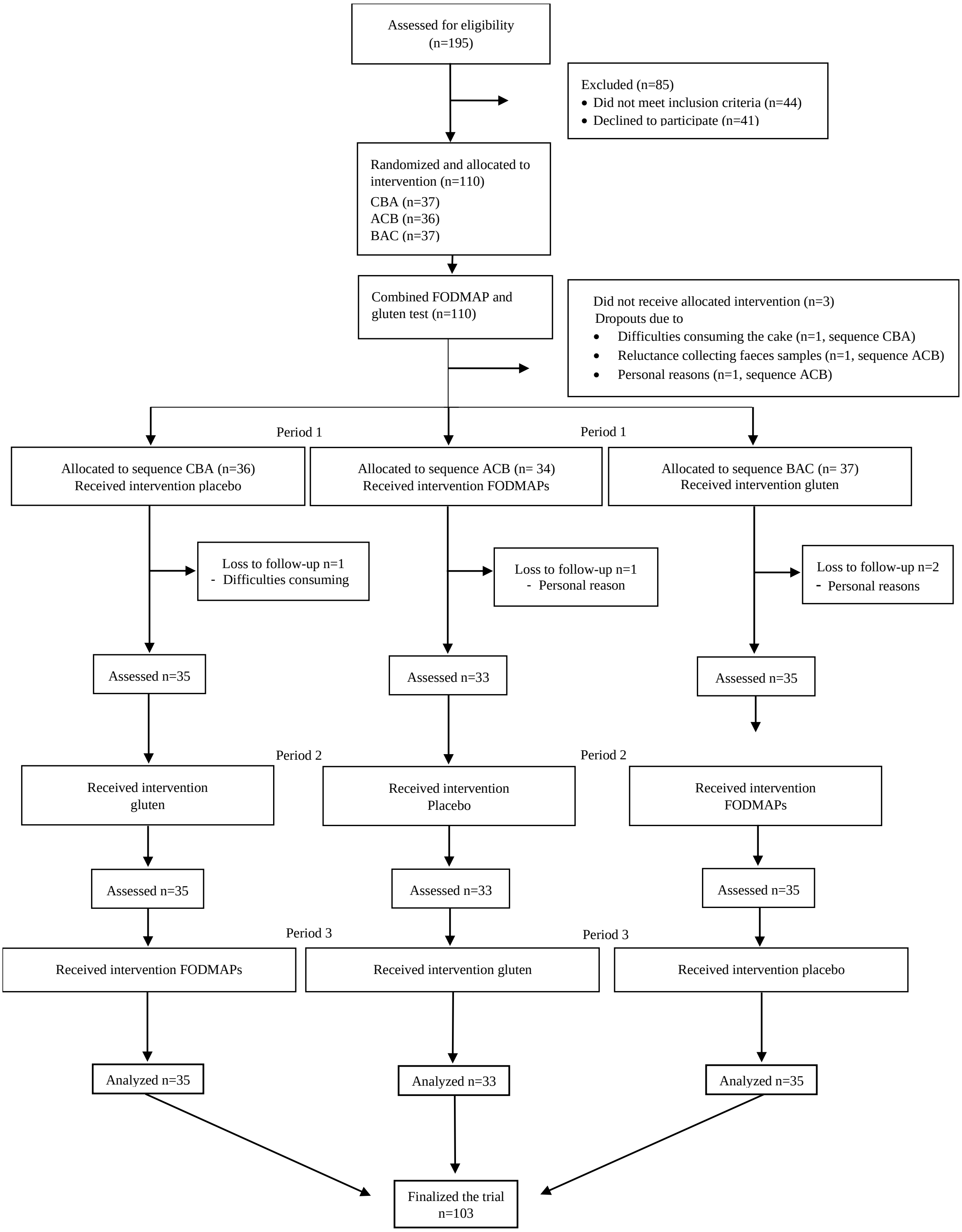

Supplementary Figure 1. Participant flow during the double-blind, randomized controlled cross-over study with FODMAPs, gluten and placebo.

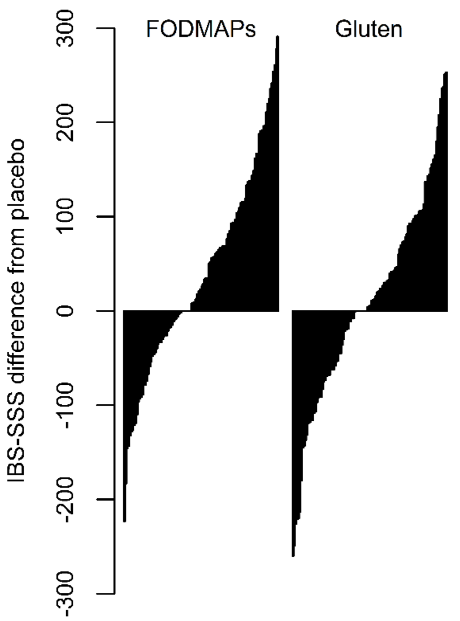

Supplementary Figure 2. The figure illustrates inter-variability in symptomatic response (total IBS-SSS score) to the interventions. For both FODMAPs and gluten, the total IBS-SSS score per participant was plotted as a difference for placebo.

Abbreviations: FODMAPs, fermentable oligo-, di-, monosaccharides and polyols; IBS-SSS, Irritable bowel syndrome severity scoring system

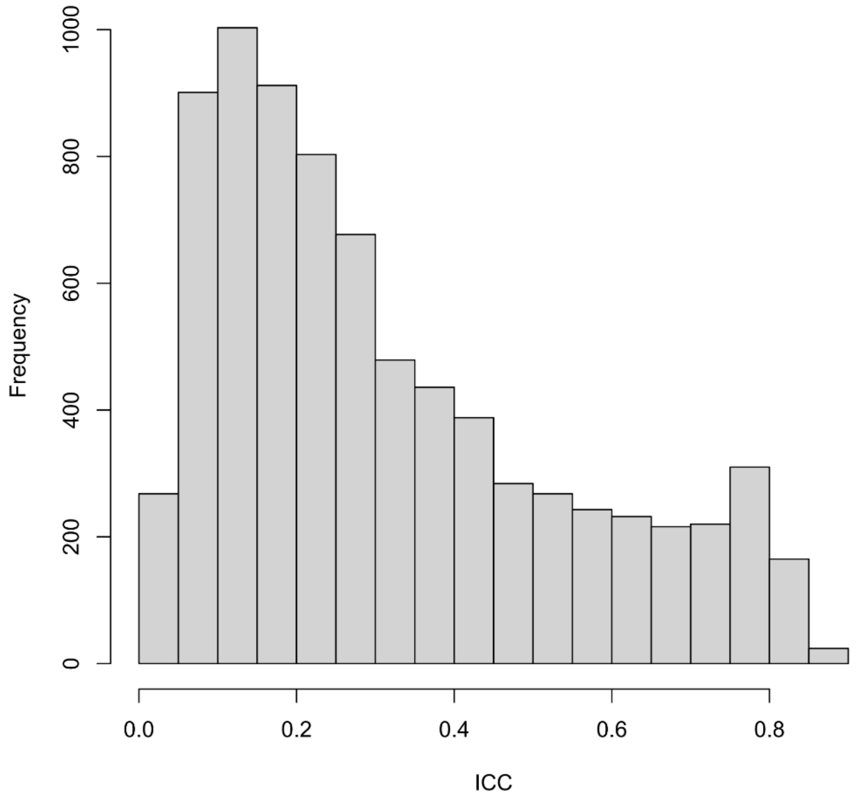

Supplementary Figure 3. A histogram over the intraclass correlation (ICC) for metabolites detected with untargeted metabolomics for the combined FODMAPs and gluten provocation test. Plasma samples were analyzed at time points -10, 0, 10, 20, 30, 90, 150, and 240 minutes.
Abbreviation: FODMAPs, fermentable oligo-, di-, monosaccharides and polyols

References

1. Smith, C. A., Want, E. J., O'Maille, G., Abagyan, R. & Siuzdak, G. XCMS: Processing mass spectrometry data for metabolite profiling using nonlinear peak alignment, matching, and identification. *Anal Chem* **78**, 779–787 (2006).

2. Libiseller, G. *et al.* IPO: A tool for automated optimization of XCMS parameters. *BMC Bioinformatics* **16**, 118 (2015).

3. Brunius, C., Shi, L. & Landberg, R. Large-scale untargeted LC-MS metabolomics data correction using between-batch feature alignment and cluster-based within-batch signal intensity drift correction. *Metabolomics* **12**, (2016).

4. Broeckling, C. D., Afsar, F. A., Neumann, S., Ben-Hur, A. & Prenni, J. E. RAMClust: A novel feature clustering method enables spectral-matching-based annotation for metabolomics data. *Anal Chem* **86**, 6812–6817 (2014).
